# Activated CD4^+^ T cells enter the splenic T-cell zone and induce autoantibody-producing germinal centers through bystander activation

**DOI:** 10.1002/eji.201343811

**Published:** 2013-11-04

**Authors:** David Banczyk, Kathrin Kalies, Lars Nachbar, Lars Bergmann, Philipp Schmidt, Ulrike Bode, Bianca Teegen, Philipp Steven, Tanja Lange, Johannes Textor, Ralf J Ludwig, Winfried Stöcker, Peter König, Eric Bell, Jürgen Westermann

**Affiliations:** 1 Center for Structural and Cell Biology in Medicine, Institute of Anatomy, University of LübeckLübeck, Germany; 2 Department of Anatomy, Medical School of HannoverHannover, Germany; 3 Euroimmun AGLübeck, Germany; 4 Department of Internal Medicine I, University of LübeckLübeck, Germany; 5 Theoretical Biology and Bioinformatics, Utrecht UniversityUtrecht, The Netherlands; 6 Department of Dermatology, University of LübeckLübeck, Germany; 7 Immunology Research Group, University of ManchesterManchester, United Kingdom

**Keywords:** Activated T cells, Development, Germinal center, Noncognate interaction, Rodent

## Abstract

CD4^+^ T (helper) cells migrate in huge numbers through lymphoid organs. However, little is known about traffic routes and kinetics of CD4^+^ T-cell subsets within different organ compartments. Such information is important because there are indications that CD4^+^ T cells may influence the function of microenvironments depending on their developmental stage. Therefore, we investigated the migration of resting (naïve), activated, and recently activated (memory) CD4^+^ T cells through the different compartments of the spleen. Resting and recently activated CD4^+^ T cells were separated from thoracic duct lymph and activated CD4^+^ T cells were generated in vitro by cross-linking the T-cell receptor and CD28. The present study shows that all three CD4^+^ T-cell subsets selectively accumulate in the T-cell zone of the spleen. However, only activated T cells induce the formation of germinal centers (GCs) and autoantibodies in rats and mice. Our results suggest that in a two-step process they first activate B cells independent of the T-cell receptor repertoire and CD40 ligand (CD154) expression. The activated B cells then form GCs whereby CD154-dependend T-cell help is needed. Thus, activated T cells may contribute to the development of autoimmune diseases by activating autoreactive B cells in an Ag-independent manner.

## Introduction

CD4^+^ T (helper) cells play a major role in the immune system. On one hand, they help macrophages respond to intracellular Ags. On the other hand, they help B cells form GCs 1,2. Germinal centers (GCs) only develop in homoeothermic organisms and facilitate high-affinity and long-lasting Ab responses against extracellular Ags 3. To fulfill their tasks, CD4^+^ T cells have to migrate between and within the tissues of the body 4,5. Resting (naïve) CD4^+^ T cells continuously leave the blood and enter secondary lymphoid organs such as spleen, LNs, tonsils, and Peyer's patches where they selectively accumulate in the T-cell zone, thereby randomly screening the microenvironment before exiting 6,7. After returning to the blood, a new round of migration begins. Resting CD4^+^ T cells bearing the appropriate T-cell receptor become activated only if they engage cognate Ag presented via MHC class II. The majority of the clonally expanded population has a short lifespan — between hours and days (summarized in 8,9). It is unclear whether activated T cells can enter the T-cell zone of secondary lymphoid tissues and whether they are able to selectively accumulate there such as resting (naïve) CD4^+^ T cells 4,5. The activated T cells may develop into different types of short-lived effector CD4^+^ T cells 10. There is broad consensus that a fraction of activated CD4^+^ T cells stabilizes as a population after expansion and contraction phases of an immune response 11 becoming recently activated (memory) CD4^+^ T cells. It is unknown how these cells migrate within tissue compartments.

Information about traffic patterns is important since functions of CD4^+^ T-cell subsets such as adhesion molecule expression, cytokine production, and proliferation rate can be influenced by the characteristic milieu of tissue compartments 12,13. Furthermore, migrating T cells are able to change the tissue milieu. For example, upon entry activated T cells increase the expression of IL-2 and IFN-γ within the splenic T-cell zone 14. This might contribute to the development of autoimmunity into which the spleen seems to be involved 1,15,16. For CD4^+^ T-cell migration the spleen plays a key role, as it harbors more mature CD4^+^ T cells than any other single organ in the body 17. Since the CD4^+^ T-cell subsets vary considerably in the expression of adhesion molecules and chemokine receptors 9,18, it must be assumed that resting (naïve), activated, and recently activated (memory) CD4^+^ T cells migrate differently through the splenic compartments (T-cell zone, B-cell zone, marginal zone, and the red pulp). For example, LFA-1 that is needed for entry into the T-cell zone is significantly higher expressed on activated and recently activated than on resting CD4^+^ T cells 19. In contrast, the chemokine receptor, CCR7, that plays a role in retaining CD4^+^ T cells in the T-cell zone is downregulated on activated compared to resting CD4^+^ T cells 9.

To test if resting (naïve), activated, and recently activated (memory) CD4^+^ T cells indeed differ in their migratory behavior, we investigated their migration through the compartments of the spleen. We show that CD4^+^ T-cell subsets are all able to selectively accumulate within the T-cell zone of the spleen, although they vary significantly in adhesion molecule and chemokine receptor expression. Within the spleen only activated CD4^+^ T cells induce the formation of autoAb-producing GCs. Our results suggest that this takes place in a two-step process. First, B cells are activated by noncognate interaction and independent of CD154 expression (CD40L). Activated B cells then proceed to form GCs, thereby requiring the presence of CD154 expressed by T cells. Thus, activated CD4^+^ T cells may contribute to the induction and aggravation of autoimmune diseases by noncognate activation of autoreactive B cells.

## Results

### CD4^+^ T cells selectively accumulate in the T-cell zone irrespective of their activation status

**Figure 1:**
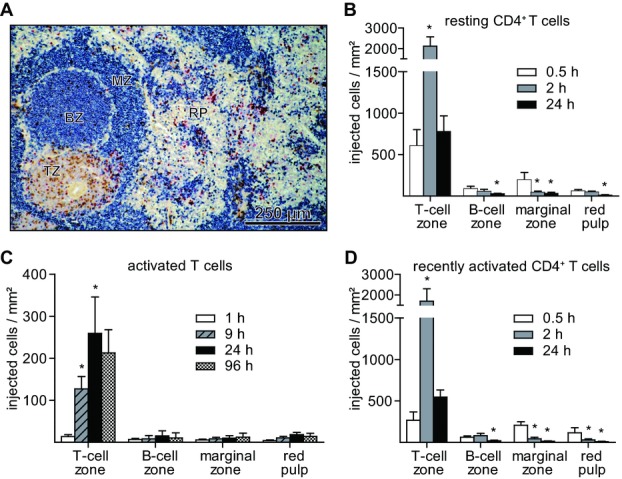
Resting (naïve), activated, and recently activated (memory) CD4^+^ T cells selectively accumulate in the T-cell zone of the rat spleen. (A) Cryosection of a rat spleen 2 h after injection of resting CD4^+^ T cells (brown, CD45.1). The different splenic compartments are visualized by staining the endogenous B cells (blue; TZ, T-cell zone; BZ, B-cell zone; MZ, marginal zone; RP, red pulp; proliferating cells, red). (B–D) Localization of (B) resting, (C) activated, or (D) recently activated CD4^+^ T cells following adoptive transfer. Data are shown as mean + SD of 4–6 (B), 6–10, (C), or 4–6 animals (D) at each interval from one experiment representative of two and three performed. *p < 0.05, difference to 0.5-h value in (B) and (D); (C) difference to the previous value, Mann–Whitney *U* test.

Resting (naïve) CD4^+^ T cells were identified in the T-cell zone (periarteriolar lymphoid sheath), B-cell zone (follicle), marginal zone, and the red pulp of the spleen in rats 20 and quantified (Fig.1A and B). Thirty minutes after injection, the majority of resting CD4^+^ T cells was localized in the T-cell and marginal zone (Fig.1B). The numbers of resting CD4^+^ T cells then declined over time in all splenic compartments except the T-cell zone. Here, their numbers reached a maximum 2 h after injection being about 30 times higher than in any other compartment of the spleen. Activated T cells, too, selectively accumulated in the T-cell zone. However, the maximum number was reached significantly later (24 h after injection) and remained much longer (for at least 72 h; Fig.1C). Surprisingly, recently activated (memory) CD4^+^ T cells regained the migration pattern of resting (naïve) CD4^+^ T cells (Fig.1D).

Since there is some uncertainty as to whether LFA-1 is involved in mediating lymphocyte entry into the spleen 21,22, we wanted to know whether it may play a role in the selective accumulation of CD4^+^ T cells in the T-cell zone. Mouse CD4^+^ T cells showed a similar migration pattern through splenic compartments as that of rat CD4^+^ T cells. Only 2 h after injection, the number of CD4^+^ T cells in the T-cell zone of mice was already about 30 times higher than that in the B-cell zone (T/B ratio: 32 ± 13; *n* = 6) and within 24 h this ratio halved (17 ± 7; *n* = 6). Importantly, CD4^+^ T cells from LFA-1-deficient animals revealed the same migration pattern through the spleen as their WT counterparts (T/B ratio 2 h: 49 ± 25, *n* = 6; T/B ratio 24 h: 20 ± 7, *n* = 6), indicating that LFA-1 is not involved in the selective accumulation of CD4^+^ T cells in the T-cell zone of spleen.

### Activated T cells induce proliferation of endogenous T and B cells and formation of GCs

**Figure 2:**
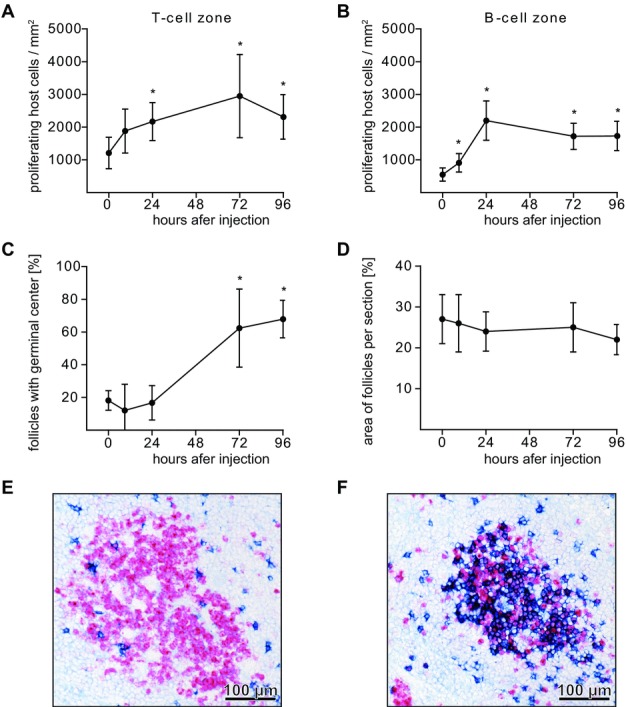
Activated T cells induce proliferation of host cells and GC formation in rat spleen. (A, B) After adoptive transfer of in vitro activated CD4^+^ T cells, Ki67-positive host cells were identified in cryosections and their numbers determined in (A) the T-cell zone and (B) the B-cell zone (excluding GCs; host cells were differentiated from the injected cells by a congenic marker). (C) The percentage of follicles harboring GCs and (D) the area of follicles per section were determined by microscopic morphometry. (A–D) Data are shown as mean ± SD of six to seven animals for each time point and are pooled from two and three experiments. *p < 0.05, difference to first value, Mann–Whitney *U* test. (E, F) After injection of in vitro activated lymphocytes (blue), (E) GCs develop (Ki67-positive cells, red), which are of host origin. (F) Only rarely they are of donor origin (blue). (E, F) Images shown are representative of more than 50 sections evaluated from two and three experiments.

Three days after injection of activated T cells, 3.5 ± 1.1% (*n* = 11) were able to incorporate BrdU while being within the splenic T-cell zone, whereas less than 0.2% of resting and recently activated CD4^+^ T cells were BrdU-positive 23. This shows that activated T cells are able to maintain their proliferative capacity for several days after injection and we asked whether they are able to induce host cell activation. One day after injection of activated T cells, the number of Ki67-positive host T cells (cells that entered the cell cycle) increased significantly and remained elevated for 3 days (Fig.2A). Surprisingly, after injection of activated T cells, the number of proliferating host B cells also increased significantly (Fig.2B) as well as the number of GCs (Fig.2C). The area of follicles per splenic section remained constant (Fig.2D), which demonstrates an absolute increase in splenic GC area. Analysis of 119 GCs showed that 93.3% were of host origin (Fig.2E) and 6.7% of donor origin (Fig.2F). Apparently, activated T cells are able to activate not only host B cells but also coinjected donor B cells. LNs and Peyer's patches did not develop GCs although activated T cells entered these tissues. In addition, after adoptive transfer of resting and recently activated CD4^+^ T cells, no GC formation in lymphoid organs was observed.

### Activated T cells induce GC formation in the spleen of mice

**Figure 3:**
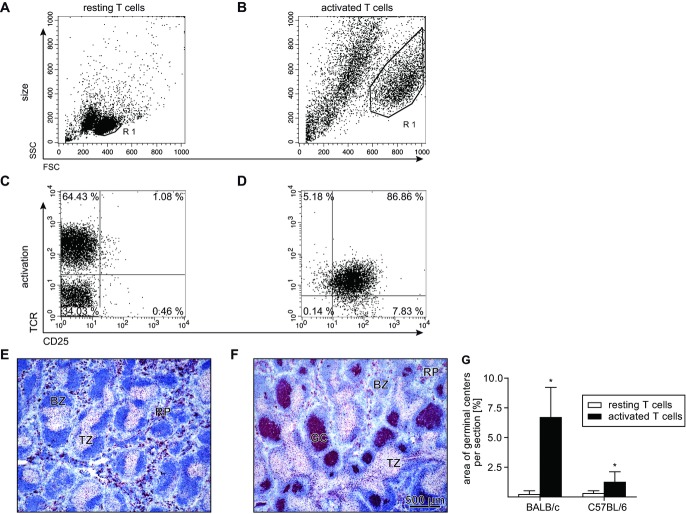
Activated T cells induce GC formation in the spleen of mice. (A–D) Cells shown in (C) and (D) are the gated cells from (A) and (B). Compared with (A, C) resting T cells, (B) activated T cells showed an increase in cell size after 3 days of in vitro stimulation with (D) an increase in CD25 expression and a decrease in T-cell receptor expression. (E, F) BALB/c spleen 6 days after adoptive transfer of either (E) resting or (F) in vitro activated T cells (Ki67-positive cells, red; B cells, blue; GC, germinal center; TZ, T-cell zone; BZ, B-cell zone; RP, red pulp). (A–F) Data shown are representative of four and five experiments performed. (G) Area of GCs per splenic section 6 days after injection of activated T cells into BALB/c and C57BL/6 mice. Data are shown as mean + SD of 18–21 animals per group, which have been obtained from four and five experiments and were then pooled. * *p* ≤ 0.001, Mann–Whitney *U* test.

**Figure 4:**
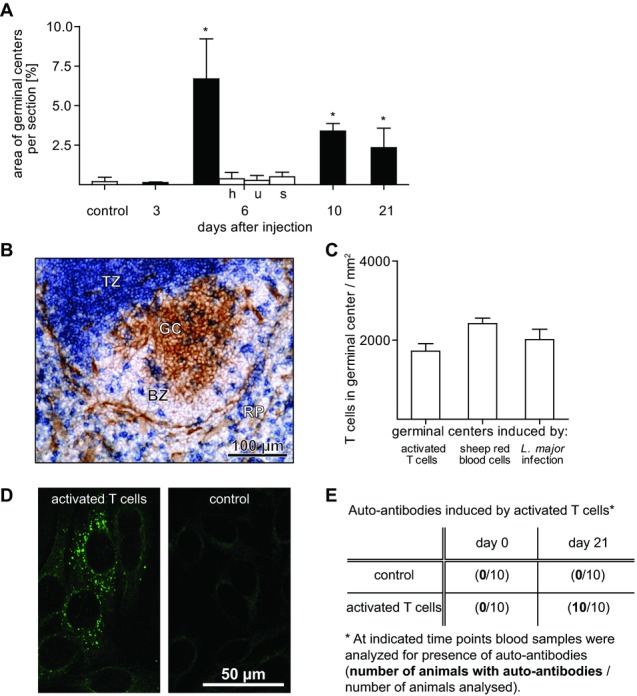
GCs induced by activated T cells are visible for more than 2 weeks, contain many T cells, and produce autoantibodies. (A) Area of GCs after injection of activated T cells (black columns, *n* = 3–18 per group; control: *n* = 30). h and u: prior to adoptive transfer, activated T cells were killed by heat (h, *n* = 4) or ultrasonic treatment (u, *n* = 4). s: only supernatant generated during T-cell activation in vitro was injected (s, *n* = 4). Data are shown as mean + SD. * *p* < 0.001, one-way ANOVA and Dunnett posthoc test. (B) Identification of T cells (CD3 positive; blue) within a GC (PNA positive; brown) in splenic tissue by immunohistochemistry 6 days after injection of activated T cells (BZ, B-cell zone; GC, germinal center; TZ, T-cell zone; RP, red pulp). (C) Quantification of T-cell numbers within GCs induced in the spleen after injection of activated T cells (*n* = 4, days 6), sheep red blood cells (*n* = 3, day 10) or in the LN after infection with *L. major* (*n* = 5, day 21). (D) Three weeks after adoptive transfer of activated T cells, the presence of IgG autoantibodies was determined using FITC-labeled goat antimouse IgG Ab. (E) Quantification of autoantibodies in the serum of mice before and 21 days after adoptive transfer of either PBS (control) or activated T cells. Data shown are representative of one and two experiments performed.

To determine whether GC formation by activated T cells also occurs in mice, T cells of mice were activated in vitro for 3 days by cross-linking CD3 and CD28. During activation T-cell size and number increased (Fig.3A, B), T-cell receptor expression was downregulated, and the vast majority of T cells became positive for CD25 (Fig.3C and D) and CD69 (>85%). After injection of activated T cells, GC formation in the spleen of mice was observed (Fig.3E and F). Quantitative evaluation of the histological sections showed that BALB/c mice developed more and larger GCs compared to C57BL/6 mice, resulting in a significant greater total area of GCs per splenic section in BALB/c mice (Fig.3G). Further studies in BALB/c mice showed that fully developed GCs were seen 6 days after injection of activated T cells, many of them being visible for up to 21 days after injection (Fig.4A). Neither activated T cells killed prior to injection (by heat or by ultrasonic treatment) nor the cytokines in the supernatant generated during the activation of T cells in vitro were able to induce GC formation upon injection (Fig.4A). On day 6, GCs induced by activated T cells harbored about 2000 T cells per millimeter square. This number was in the same range as for T cell dependent GCs induced by injection of sheep red blood cells or during *Leishmania major* infection (Fig.4B and C). Furthermore, analysis of the serum showed the presence of autoantibodies against cytoplasmic Ags of human epithelial 2 cells (Fig.4D) that developed in ten of ten animals 21 days after the injection of activated T cells but in none of the control animals (Fig.4E). Such autoantibodies were not detected at day 6 (control: 0/4; activated T cells: 0/6). Together, this strongly suggests that injection of activated T cells induces the formation of T cell dependent GCs that produce autoantibodies.

### GC formation does not depend on the T-cell receptor repertoire of the activated T cells

**Figure 5:**
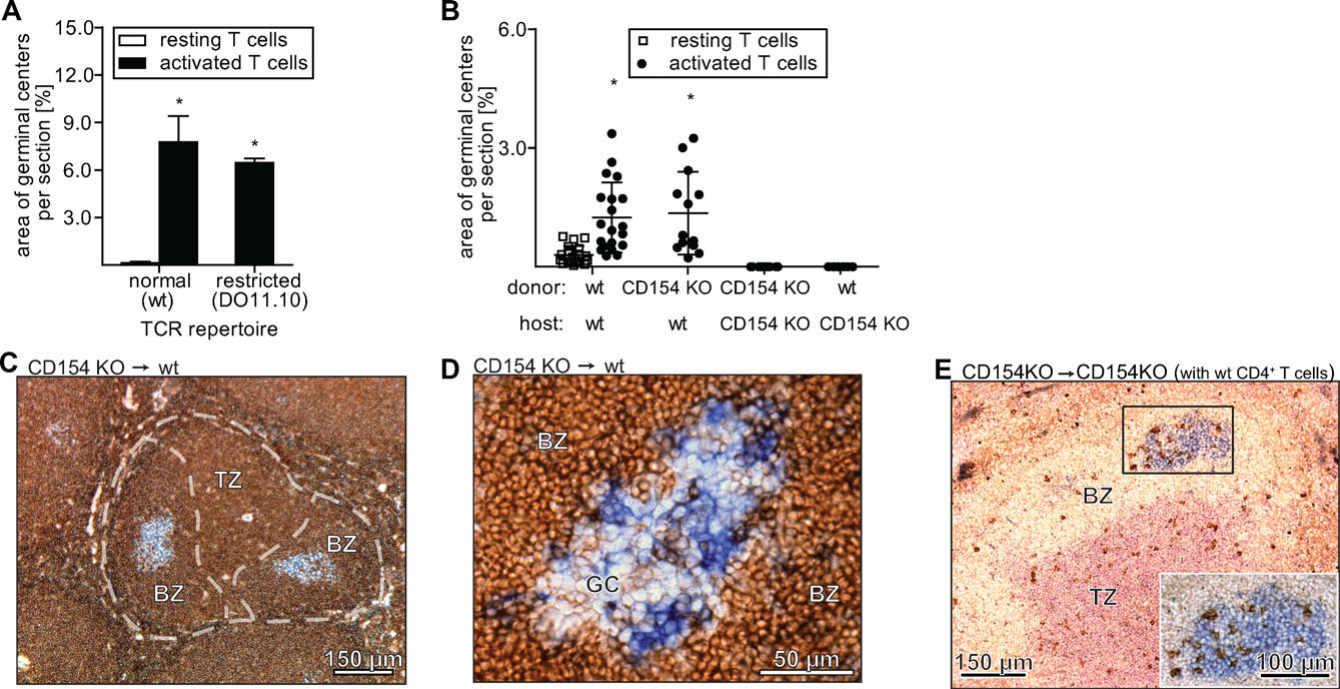
Analysis of T-cell receptor repertoire and CD154 in regulating the formation of GCs induced by activated T cells. (A) Splenic GC size in recipients 6 days after injection of activated T cells obtained from WT or DO11.10 BALB/c mice (*n* = 4 per group). Data are shown as mean + SD of the indicated number of mice from one experiment. (B) Splenic GC size in WT recipients 6 days after injection of resting (*n* = 20) or activated T cells from WT (*n* = 20) or CD154-deficient C57BL/6 donors (*n* = 13), and in CD154-deficient recipients after injection of activated T cells from CD154-deficient (*n* = 12) and WT donors (*n* = 6). Each symbol represents an individual mouse and the data are shown as mean + SD. Data shown are pooled from two and three experiments. * *p* < 0.001, one-way ANOVA and Dunnett posthoc test. (C) B cells (CD45.2, B220, blue) that were activated in vitro by T cells from CD154-deficient mice formed GCs after injection into a WT host (CD45.1, brown). (D) After in vitro activation and injection into a WT host (CD45.1, brown), CD4^+^ T cells from CD154-deficient mice were found in GCs 6 days later (CD45.2, blue). (E) T cells from CD154-deficient donors (CD45.2) were activated and injected into a CD154-deficient host (CD45.2) that had received resting CD4^+^ T cells from WT donors (CD45.1). A GC had developed in a CD154-deficient host (PNA, blue; endogenous T cells: CD3, red; WT CD4^+^ T cells: CD45.1, brown; wt, wild-type; CD154 KO, deficient; BZ, B-cell zone; TZ, T-cell zone). (C–E) Data shown are representative of 38 sections analyzed from six and seven animals.

After activation by APCs, CD4^+^ T cells activate B cells to induce GC formation. This occurs by cognate interaction of the T-cell receptor with MHC class II/peptide complex expressed by B cells 24,25. To find out whether in vitro activated T cells also communicate with B cells by this specific interaction, CD4^+^ T cells from DO11.10 mice were used 26. About 80% of the CD4^+^ T cells of these mice express a T-cell receptor specific for a peptide of ovalbumin 27. Thus, compared with WT T cells, transgenic T cells have a severely compromised T-cell receptor repertoire. Thus, if the interaction of in vitro activated CD4^+^ T cells with B cells is MHC class II dependent, one would expect significantly less GCs to be formed after injection of activated CD4^+^ T cells from DO11.10 mice. Surprisingly, activated transgenic T cells induced GC formation as efficiently as activated T cells from WT animals (Fig.5A). This indicates that B-cell activation probably occurs by MHC class II independent mechanisms.

### Activation of B cells is CD154-independent whereas formation of GCs is CD154-dependent

The interaction between CD40 ligand (CD154; expressed by CD4^+^ T cells) and CD40 (expressed by B cells) is necessary for Ag-induced GC formation 28. To find out whether activated T cells need CD154 to induce GC formation in vivo, CD154-deficient T cells were activated in vitro and injected into WT animals. Surprisingly, CD154-deficient T cells were able to induce GC formation (Fig.5B). In contrast, when activated T cells from CD154-deficient mice were injected into CD154-deficient mice, no GC formation was observed (Fig.5B). In addition, activated WT T cells were also unable to induce GC formation after adoptive transfer into CD154-deficient hosts (Fig.5B). The observation that both WT and CD154-deficient CD4^+^ T cells were able to induce GCs in WT hosts but neither WT nor CD154-deficient CD4^+^ T cells could do so in a CD154-deficient milieu suggests a two-step process of GC formation. In the first step (B-cell activation), T cells of donor origin are involved, which do not need to express CD154, whereas during the second step (GC formation) the presence of CD154-expressing host T cells is required.

To determine if the initial step of GC formation can be induced by CD154-deficient T cells, WT B cells were cultured for 3 days in vitro in the presence of activated CD154-deficient CD4^+^ T cells. Then, the B cells were injected into congenic WT animals. Indeed, in six of six animals GCs were observed formed by the injected B cells (Fig.5C, blue), demonstrating that CD154-deficient CD4^+^ T cells can induce B cells to build GCs in vivo. In addition, donor CD4^+^ T cells were found within the induced GCs (Fig.5D, blue). We next evaluated whether the second step in GC formation was dependent on CD154 expressing T cells. In a first experiment, CD154-deficient CD4^+^ T cells were activated in vitro and injected into CD154-deficient hosts. As expected, no GC formation was observed (Fig.5B). However, if resting CD4^+^ T cells from congenic WT mice (5 × 10^6^; purity: >98%) were injected 2 days before adoptive transfer of activated CD154-deficient T cells, GC formation was observed in CD154-deficient hosts in 3 of 7 animals (Fig.5E) whereas not a single GC was found in 18 of 18 CD154-deficient control mice without prior adoptive transfer of WT CD4^+^ T cells (Fig.5B). In addition, WT CD4^+^ T cells accumulated within the splenic GCs formed in CD154-deficient hosts (Fig.5E).

Together, our data demonstrate that activated CD4^+^ T cells are able to induce GC formation in vivo in a two-step process. During the first step, activated CD4^+^ T cells activate B cells by MHC class II and CD154-independent mechanisms. In the second step, activated B cells form GCs only if CD154 is provided by CD4^+^ T cells.

## Discussion

It is well established that resting (naïve) CD4^+^ T cells are selectively enriched in the splenic T-cell zone, their numbers being up to 30 times higher than in the other splenic compartments 29. We show that LFA-1 is not required for selective accumulation within the T-cell zone since LFA-1-deficient CD4^+^ T cells are as efficient as WT CD4^+^ T cells in this process. This is in agreement with earlier studies reporting that blocking of LFA-1 had none or only very minor effects on T-cell entry into the splenic T-cell zone 22. We extend these observations here by showing that LFA-1 is also not required for selective concentration of resting CD4^+^ T cells within the T-cell zone. Furthermore, activated and recently activated (memory) CD4^+^ T cells, too, selectively accumulate in the splenic T-cell zone. This is surprising because the chemokine receptor, CCR7, that facilitates T-cell migration into and within the T-cell zone 30 is upregulated on resting, downregulated on activated, and intermediately expressed on recently activated CD4^+^ T cells 9,18, and yet all three subsets selectively accumulate within the T-cell zone. This indicates that the CCR7 pathway is not sufficient to explain this process and suggests that major mechanisms facilitating the enrichment of CD4^+^ T-cell subsets in the T-cell zone still need to be identified 4,31.

We show for the first time that activated T cells have the ability to induce both, the proliferation of endogenous T and B cells and the formation of GCs. Although cytokines and fragments of activated T cells are capable of inducing B-cell activation and proliferation in vitro 32, the present study demonstrates that neither cell fragments nor cytokine-containing supernatants are able to induce GC formation in vivo — intact activated T cells are required. Several lines of evidence suggest that these are T-cell dependent GCs: (i) they exist for several weeks 33, (ii) they harbor as many T cells as the T cell dependent GCs induced after immunization with sheep red blood cells or after infection with *L. major* (Fig.4C), and (iii) injection of activated T cells into CD154-deficient hosts that are able to develop T-cell independent but not T-cell dependent GCs 28 does not lead to formation of GCs in the spleen (Fig.5B). Interestingly, when activated T cells become recently activated (memory) CD4^+^ T cells, they lose the ability to induce GC formation in the spleen probably because they now much more resemble resting than activated T cells (present study and 18). Furthermore, LNs and Peyer's patches fail to develop GCs although activated T cells enter these tissues. This may be due to the lower number of activated T cells accumulating in the T-cell zone of LNs — 150 per millimeter square 1 day after injection 12 — compared with 300 per millimeter square in the T-cell zone of the spleen. In addition, the cytokine milieu within the T-cell zone may also play a role since it differs significantly between spleen and LNs 14.

Current thinking suggests that the formation of T cell dependent GCs is initiated by APCs that activate CD4^+^ T cells in the T-cell zone of lymphoid organs such as LNs and spleen 25,34. These cells then migrate to the T–B border 35 where they activate B cells arriving there after recognizing Ag in the B-cell zone 36. The subsequent activation of B cells in vivo depends on both the interaction of the T-cell receptor with the MHC class II/peptide complex and the interaction of CD40 ligand (CD154) expressed by CD4^+^ T cells with CD40 expressed by B cells. Through these interactions cognate B cells are licensed to form GCs 24,25. Our study now shows that activated T cells with either a diverse or a severely restricted T-cell receptor repertoire activate B cells to form GCs with the same efficiency. If the T-cell receptor is involved in the activation of B cells, CD4^+^ T cells with a restricted repertoire should have performed less efficiently for the chance of cognate T-cell receptor and MHC class II interaction would be severely reduced; but this was not the case, thus indicating that there are molecular mechanisms leading to B-cell activation that are independent of the cognate interaction between T-cell receptor and the MHC class II/peptide complex. However, to finally prove this mechanism, it must be directly shown that the T-cell receptor/MHC class II interaction is dispensable for the B-cell activation observed in the present study. In addition, a recent study shows that the continuous presence of regulatory T cells is necessary to prevent B cells from forming GCs and producing autoantibodies 37. Thus, the effects observed in the present study might be caused by an inhibitory effect of activated T cells on such regulatory T cells. This conclusion is supported by the observation of a significant increase in IL-10 expression in the splenic T-cell and B-cell zone 9 h after injection of activated T cells 14.

The present study confirms that the presence of CD154 is necessary for the development of T cell dependent GCs 2 and for the first time identifies the step in GC formation during which CD154 is needed. We show that CD154 expression is not necessary for B-cell activation. Activated T cells obtained from CD154-deficient donors are as efficient as WT cells for activating B cells and inducing GCs (Fig.5B). In contrast, the presence of CD154 is needed for formation of GCs. When activated T cells from CD154-deficient donors are adoptively transferred into CD154-deficient hosts, no GCs develop, although B cells are activated (Fig.5B). However, GCs develop when prior to injection of activated T cells (from CD154-deficient donors) WT CD4^+^ T cells are adoptively transferred. Interestingly, these WT CD4^+^ T cells seem to be attracted to evolving GCs in CD154-deficient hosts since more than ten CD4^+^ T cells were observed at day 6 in a histological section of an evolving GC (Fig.5E). In contrast, in established GCs, on average less than one CD4^+^ T cell per section was found within 48 h after injection 19. Preferential migration into follicles and GCs is a key feature of follicular helper T (Tfh) cells (18). Thus, adoptive transfer of activated CD4^+^ T cells from CD154-deficient donors probably induces the development of Tfh cells among the resting WT CD4^+^ T cells of the host. This conclusion is supported by a study showing that increased numbers of Tfh cells lead to GC formation and autoAb production in the absence of foreign Ag 38, effects also observed in the present study.

The induction of GCs in the spleen through noncognate activation is of clinical relevance. Many studies report an association between infections and the development of autoimmune diseases 39–41, and recently evidence was summarized suggesting a correlation between GC formation and the development of autoimmune diseases such as type 1 diabetes and rheumatoid arthritis 1,42. The present study shows for the first time in an animal model that adoptively transferred activated T cells that make up about 1–2% of circulating lymphocytes in the blood 23 — enter the spleen and induce GC formation. Similar numbers of activated CD4^+^ T cells in the blood are often observed in humans during bacterial 43 and viral infections 44,45, and during super-Ag induced immune responses 46. Thus, it conceivable that in humans, also, activated T cells induce splenic B cells to form GCs. Since in healthy humans and mice about 10% of the B cells are autoreactive 47,48, it seems likely that autoantibodies could develop. Indeed, we demonstrate in our study that injection of activated T cells induces the development of IgG autoantibodies in all animals investigated. Although not formally proven, the late appearance of the autoantibodies in the serum (after day 6) makes it very likely that they originate from the newly formed GCs 49. Thus, during severe infections cognate interactions lose their importance whereas the CD154–CD40 pathway still is instrumental for GC formation, underlining its role as therapeutic target in the treatment of autoimmune diseases 50 that in the developed countries represent the third leading causes for morbidity and mortality after cancer and heart disease 40.

## Materials and methods

### Animals

Male congenic rats from the inbred PVG.7A (RT7a) and PVG.7B (RT7b) strains 51 and the inbred LEW/Ztm (RT.7a) and LEW.7B/Won (RT.7b) strains were used 12. BALB/c, C57BL/6, congenic C57BL/6Ly5.1, DO11.10 mice (C.Cg-Tg(DO11.10)10Dlo/J), and CD154 (CD40L) deficient mice (B6; 129S2-Cd40lgtm1Imx/J; provided by D. Gray, Edinburgh, GB) were used. Permission for the animal experiments was issued by the Animal Care and Use Committee (Kiel, Germany, V 312-72241.122-1/55-5/09).

### Adoptive transfer of resting and recently activated CD4^+^ T cells

To obtain resting CD4^+^ T cells (naïve; before Ag encounter, small cell size, CD45RC-positive, CD62L high, CD44 low) and recently activated CD4^+^ T cells (memory; after activation, small cell size again, still CD45RC-negative, CD62L low, CD44 high), congenic rats from the inbred PVG.7A (RT7a) and PVG.7B (RT7b) strains were used and the CD4^+^ T-cell subsets purified as described 51. A total of 2 × 10^7^ CD4^+^ T cells (either resting or recently activated) were injected i.v. To obtain CD4^+^ T cells from mice, single-cell suspensions were prepared from pooled LNs. CD4^+^ T cells were isolated using negative selection by magnetic cell separation (MACS®, MiltenyiBiotec, Bergisch Gladbach, Germany). Purity and viability (determined by propidium iodide staining; BD Biosciences) of the enriched CD4^+^ T-cell population was always >95%. A total of 5 × 10^6^ CD4^+^ T cells (CD45.1) were injected i.v. into congenic recipients (CD45.2) and the spleens were removed as indicated above. The migration of LFA-1 (CD11a/CD18) deficient CD4^+^ T cells (provided by N. Hogg, London, GB) was analyzed as described 21.

### Adoptive transfer of activated CD4^+^ T cells

Activated CD4^+^ T cells were generated by stimulating LN lymphocytes in vitro via cross-linking the T-cell receptor and CD28 for 3 days 12. Rats from the standard inbred strain LEW/Ztm (RT.7a) and the congenic strain LEW.7B/Won (RT.7b) were used 12. Cell suspensions were prepared from LNs of LEW.7B rat. T cells were stimulated via the αβ T-cell receptor (Ab R73) and CD28 (Ab JJ319 provided by T. Hünig, Würzburg, Germany) for 3 days either in the presence or in the absence of BrdU as described 12. After 3 days of in vitro stimulation, the cell suspensions contained 92 ± 8% activated lymphocytes as judged by staining with BrdU; 81 ± 4% of the BrdU positive cells were characterized as T cells and 20 ± 4.0% as B cells 12. Dead cells and cell debris were always removed by centrifugation through a serum cushion. A total of 7 × 10^7^ lymphocytes were injected i.v. over 2 min into the recipients (RT7a).

To activate murine CD4^+^ T cells, 1 × 10^7^ cells were cultured in dishes that were coated with 1 mg anti-CD3 (BD Biosciences) and 3 mg anti-CD28 (provided by T. Hünig, Würzburg, Germany). After 3 days cells were harvested and washed. A total of 1 × 10^7^ lymphocytes were injected i.v. As controls, freshly isolated, activated cells that were killed by heat (90°C for 45 min) or ultrasonic treatment, and the culture supernatant (200 μL) were injected.

### Identification of injected cells in splenic compartments

To visualize injected CD4^+^ T cells within the different compartments of the rat and mouse spleen, immunohistochemistry was performed as described 12,20. To identify T cells that entered the cell cycle, splenic tissue was stained for the rat homologue of the Ki-67 Ag (monoclonal Ab MIB-5, Dako) as described 20.

### Induction of T cell dependent GCs

SRBC and *L. major* parasites were injected as described 14,52. After 10 days (spleen) and 21 days (draining LN), T cells within GCs were identified by immunohistochemistry and quantitatively determined 19.

### Identification of autoantibodies

Screening for autoantibodies was performed as described 53,54. Serum samples from mice before and 6 and 21 days after injection of activated T cells were diluted 1/10, 1/32, and 1/100, and then incubated on human epithelial 2 cells, primate liver, rat kidney, and rat stomach. Binding of IgG autoantibodies was determined using FITC-labeled goat antimouse IgG Ab 53.

### Statistical analysis

For picture acquisition, an Axiophot microscope with an Axiocam HR camera was used (Carl Zeiss, Göttingen, Germany). The open source software ImageJ was used to measure the size of GCs. Means and SDs were determined and differences were analyzed using the Mann–Whitney *U* test, Wilcoxon matched pairs signed-rank test, or one-way ANOVA and Dunnett posthoc test (SPSS for Windows and GraphPadPrism 5.0 software; GraphPad Software, Inc., La Jolla, CA, USA).

## Abbreviations

BrdU: 5-bromo-2′-deoxyuridine
GC: germinal center
LFA-1: lymphocyte function-associated antigen 1
Tfh: follicular helper T cell
